# Impact of telitacicept on gonadal function in adult female patients with systemic lupus erythematosus: a prospective cohort study

**DOI:** 10.3389/fimmu.2025.1610136

**Published:** 2025-09-30

**Authors:** Ziyan Zhang, Qianni Zeng, Yabin Wen, Yuqing Zhou, Lihua Zhang

**Affiliations:** ^1^ The First-Affiliated Hospital of Hunan Normal University, Hunan Provincial People’s Hospital, Changsha, China; ^2^ Medical Science, Immunology and Pathology, The University of Sydney, Sydney, NSW, Australia; ^3^ Department of Rheumatology and Immunology, The First-Affiliated Hospital of Hunan Normal University (Hunan Provincial People’s Hospital), Changsha, China

**Keywords:** telitacicept, systemic lupus erythematosus, gonadal function, prospective, cohort

## Abstract

**Background/Aim:**

Telitacicept has shown promise in disease control of systemic lupus erythematosus (SLE). This study aimed to evaluate the impact of telitacicept on gonadal function in adult female patients with SLE.

**Methods:**

In this prospective cohort study, adult female SLE patients aged 18 to 45 years were included and divided into telitacicept and non-telitacicept group. Hormonal levels of estradiol (E2), follicle-stimulating hormone (FSH), luteinizing hormone (LH), prolactin (PRL), and anti-Müllerian hormone (AMH) were measured at baseline, month 1, 3, and 6 post-treatment. Generalized estimating equations adjusting for baseline confounders was used.

**Results:**

A total of 78 patients were included, with 38 in the telitacicept group and 40 in the non-telitacicept group. Telitacicept significantly decreased PRL and LH levels (both adjusted P_time_<0.001), with greater reduction compared to non-telitacicept treatment (adjusted P_group_=0.001 and <0.001, respectively). In the multivariate logistic regression, telitacicept treatment was associated with a significantly lower incidence of abnormal PRL levels at month 6 (odds ratio=0.138, 95% confidence interval: 0.036-0.527, P = 0.004). The levels of AMH and E2 were increased and the levels of FSH were decreased (all adjusted P_time_<0.05), while the changes of AMH, E2 and FSH levels were similar between the two groups (all adjusted P_group_>0.05). SLE Disease Activity Index scores were significantly lower with telitacicept compared to non-telitacicept treatment at month 1, 3 and 6 post-treatment (all P<0.05). The incidence of adverse events was similar between the two groups.

**Conclusion:**

Telitacicept demonstrates significant benefits in improving gonadal function and controlling disease activity in female SLE patients.

## 1 Introduction

Systemic lupus erythematosus (SLE) is a chronic autoimmune disease with a global prevalence ranging from 20 to 150 cases per 100,000 individuals (1, 2), affecting women of childbearing age (3). SLE presents with a wide spectrum of clinical manifestations, including but not limited to, dermatological, musculoskeletal, renal, and neurological involvement, leading to significant morbidity and mortality (4). The disease burden is profound, characterized by a high prevalence of comorbidities, increased healthcare utilization, and diminished quality of life (5). Patients with SLE often experience recurrent flares and require long-term immunosuppressive therapy, which further contributes to the overall disease burden by increasing the risk of infections and other treatment-related complications (6).

SLE can significantly impact gonadal function, leading to clinical consequences such as menstrual irregularities, diminished ovarian reserve, and infertility (7, 8). The pathophysiology involves chronic inflammation and autoantibody production, which can directly damage ovarian tissue and disrupt endocrine function. Along with its systemic involvement, which can exacerbate gonadal dysfunction, it poses substantial challenges to fertility management in affected women (9). Studies have shown that SLE can lead to reduced anti-Müllerian hormone (AMH) levels, indicating a compromised ovarian reserve, even in the absence of cytotoxic therapy (10). In addition, the traditional therapeutic regimen for SLE might impact on the gonadal function. Corticosteroids, while essential for controlling disease activity, have also been associated with alterations in sex hormone levels, further complicating reproductive health management (11). Cyclophosphamide, a potent immunosuppressant, is known to cause gonadotoxicity, resulting in decreased AMH levels and increased risk of infertility (12), though low-dose cyclophosphamide-containing regimens, such as the Euro-Lupus protocol, have been developed to mitigate these risks (13). As the introduce of novel therapies in treating SLE, the impact of these regimens on gonadal function needs to be verified.

Telitacicept, a novel fusion protein that inhibits B lymphocyte stimulator (BLyS) and a proliferation-inducing ligand (APRIL), has shown promising results in the treatment of SLE (14). Previous studies, including phase 2b (15) and phase 3 clinical trials (16), have demonstrated the efficacy and safety of telitacicept in reducing disease activity and improving clinical outcomes in patients with active SLE. The phase 2b study noted 11 pregnancies in the telitacicept group during the trial, while no pregnancies occurred in the placebo group (15). The mechanisms by which telitacicept may improve gonadal function in female SLE patients include its ability to modulate immune responses and reduce systemic inflammation, which are known contributors to gonadal dysfunction (14). In addition, a case report described a successful pregnancy in a woman with IgA nephropathy treated with telitacicept, highlighting its potential as a safe and effective treatment option for women of childbearing age who wish to conceive (17). Nevertheless, the evidence examining the effect of telitacicept on the gonadal function is lacking.

Therefore, this prospective cohort study aims to compare the gonadal function among female SLE patients treated with or without telitacicept, so as to fill the gap and gain comprehensive knowledge of the possible clinical benefit from telitacicept.

## 2 Methods

### 2.1 Study design and patients

This prospective, observational, real-world study included patients with SLE who visited the Rheumatology and Immunology Department of our hospital from October 2021 to February 2023. The study received ethical approval from the Ethics Committee of the First-Affiliated Hospital of Hunan Normal University (Hunan Provincial People’s Hospital), and all patients provided written informed consent prior to participation.

Adult women aged 18 to 45 years, with a diagnosis of SLE according to the 1997 American College of Rheumatology (ACR) criteria and moderate to severe disease activity, defined by a SLE Disease Activity Index (SLEDAI) score of 7–12 or >12, were included. Patients received conventional therapy for SLE, including glucocorticoids, antimalarial drugs, and other immunosuppressants were included, with or without the addition of telitacicept. Patients received cyclophosphamide and tripterygium glycosides were excluded due to the known gonadotoxicity. Patients diagnosed with hypothyroidism or antiphospholipid syndrome were excluded due to possible effect on gonadal function.

Other exclusion criteria included the presence of serious complications such as infection, malignancy, or cachexia; concurrent ovarian or uterine disease, pregnancy, or lactation.

### 2.2 Treatment

Patients were divided into telitacicept group, consisted of those who received telitacicept in combination with conventional therapy, and non-telitacicept group, consisted of those who received only conventional therapy. Treatment regimens were tailored according to individual patient needs and administered based on the clinical judgment of the treating physicians, independent of the study objectives due to the real-world nature.

### 2.3 Data collection and outcomes

Demographics, baseline disease characteristics, age at menarche, previous pregnancies, and history of abortion were extracted from the medical records.

To evaluate the change in gonadal function, levels of estradiol (E2), follicle-stimulating hormone (FSH), luteinizing hormone (LH), prolactin (PRL), and AMH were measured. Venous whole blood samples (3 mL) were collected from all enrolled patients in a fasting state on Day 3 of their natural menstrual cycle prior to treatment and at 1, 3, and 6 months post-treatment. E2, FSH, LH, and PRL levels were analyzed using the I2000 fully-automatic chemiluminescence immune analyzer (Abbott Co.), while AMH levels were measured with the QD-S2000 fully-automatic fluorescence immune analyzer (Nanjing Vazyme Medical Technology Co., Ltd.). The normal reference ranges for these hormones were: E2 21–251 pg/mL, FSH 3.03-8.08 IU/mL, LH 1.80-11.78 IU/mL, PRL 5.18-26.53 ng/mL, and AMH 1.18-9.16 ng/mL.

Clinical efficacy was assessed by disease activity (measured by SLEDAI score), anti-double-stranded DNA (dsDNA) antibody, platelet count, white blood cell (WBC) count, complement C3 and C4 levels, IgG levels, and 24-hour urine protein excretion. The normal ranges for WBC and platelets were 3.69-9.16×10^9^/L and 101-320×10^9^/L, respectively. Immunological parameters were determined using a fully-automatic biochemical analyzer. Changes in the doses of glucocorticoids, mycophenolate mofetil (MMF), and hydroxychloroquine were compared before and after treatment within each group. Adverse events (AEs) were recorded.

### 2.4 Statistical analysis

Statistical analyses were conducted using R software (version 4.2.3). Continuous data were summarized as mean ± standard deviation (SD) or median with interquartile range (IQR), while categorical data were presented as frequency and percentage. For between-group comparisons of continuous variables, independent Student’s t-tests or Mann-Whitney U tests were employed. Pre- and post-treatment comparisons were analyzed using paired t-tests or Wilcoxon signed-rank tests. Categorical variables were compared between groups using chi-square tests. Repeated measures data were analyzed using generalized estimating equations (GEE), adjusting for baseline factors including age, disease duration, age at menarche, previous pregnancies, and history of abortion. Pairwise comparisons within groups over time were adjusted using the Bonferroni method. To identify factors associated with abnormal sex hormone levels, multivariate stepwise logistic regression models were used and variables with P<0.05 in univariate analyses were included in the multivariate analysis. P values less than 0.05 were considered statistically significant.

## 3 Results

### 3.1 Baseline characteristics

A total of 78 patients were included in this study, with 38 in the telitacicept group and 40 in the non-telitacicept group. The mean age of patients was 29.7 ± 7.3 years in the telitacicept group and 30.5 ± 7.0 years in the non-telitacicept group (P = 0.604). The median disease duration was similar between the groups, with 175.5 days (IQR: 159.3, 214.5) in the telitacicept group and 176.0 days (IQR: 163.0, 215.3) in the non-telitacicept group (P = 0.803). The median age at menarche was 13.5 years (IQR: 12.0, 14.0) in the telitacicept group and 13.0 years (IQR: 12.0, 14.0) in the non-telitacicept group (P = 0.720). Previous miscarriages were reported in 26.3% of patients in the telitacicept group and 35.0% in the non-telitacicept group (P = 0.406). Previous pregnancies were noted in 60.5% and 67.5% of patients in the telitacicept and non-telitacicept groups, respectively (P = 0.521) (**Table 1**).

**Table 1:** Baseline characteristics of patients.

Variables	Telitacicept (n=38)	Non-telitacicept (n=40)	*P*
Age, years, mean ± SD	29.7 ± 7.3	30.5 ± 7.0	0.604
<30	21 (55.3)	18 (45.0)	0.365
≥30	17 (44.7)	22 (55.0)	
Disease course, days, median (IQR)	175.5 (159.3, 214.5)	176.0 (163.0, 215.3)	0.803
<175.5	19 (50.0)	20 (50.0)	>0.999
≥175.5	19 (50.0)	20 (50.0)	
Menarche age, years, median (IQR)	13.5 (12.0, 14.0)	13.0 (12.0, 14.0)	0.720
Previous miscarriages, n (%)	10 (26.3)	14 (35.0)	0.406
Previous pregnancies, n (%)	23 (60.5)	27 (67.5)	0.521
Sex hormones, mean ± SD			
E2, pg/ml	187.68 ± 77.00	168.40 ± 71.66	0.447
FSH, mIU/ml	5.93 ± 3.14	6.69 ± 4.71	0.285
LH, mIU/ml	8.88 ± 2.07	8.94 ± 2.22	0.899
PRL, ng/ml	42.36 ± 14.67	41.50 ± 15.12	0.646
AMH, ng/ml	1.35 ± 1.66	1.01 ± 0.73	0.542
SLEDAI, mean ± SD	10.92 ± 0.91	11.05 ± 1.13	0.466
dsDNA antibody positive, n (%)	32 (84.2)	36 (90.0)	0.455
24-hour urine protein, g/24h, mean ± SD	0.28 ± 0.36	0.16 ± 0.08	0.397
Glucocorticoid dose, mg/d, mean ± SD	26.53 ± 4.58	25.48 ± 4.18	0.246
MMF dose, g/d, mean ± SD	1.36 ± 0.23	1.43 ± 0.18	0.224
Hydroxychloroquine dose, g/d, mean ± SD	0.39 ± 0.03	0.38 ± 0.04	0.557

SD, standard deviation; IQR, interquartile range; E2, estradiol; FSH, follicle-stimulating hormone; LH, luteinizing hormone; PRL, prolactin; AMH, anti-Müllerian hormone; SLEDAI, Systemic Lupus Erythematosus Disease Activity Index; dsDNA, double-stranded DNA; MMF, mycophenolate mofetil.

### 3.2 Sex hormone levels

**Figure 1:**
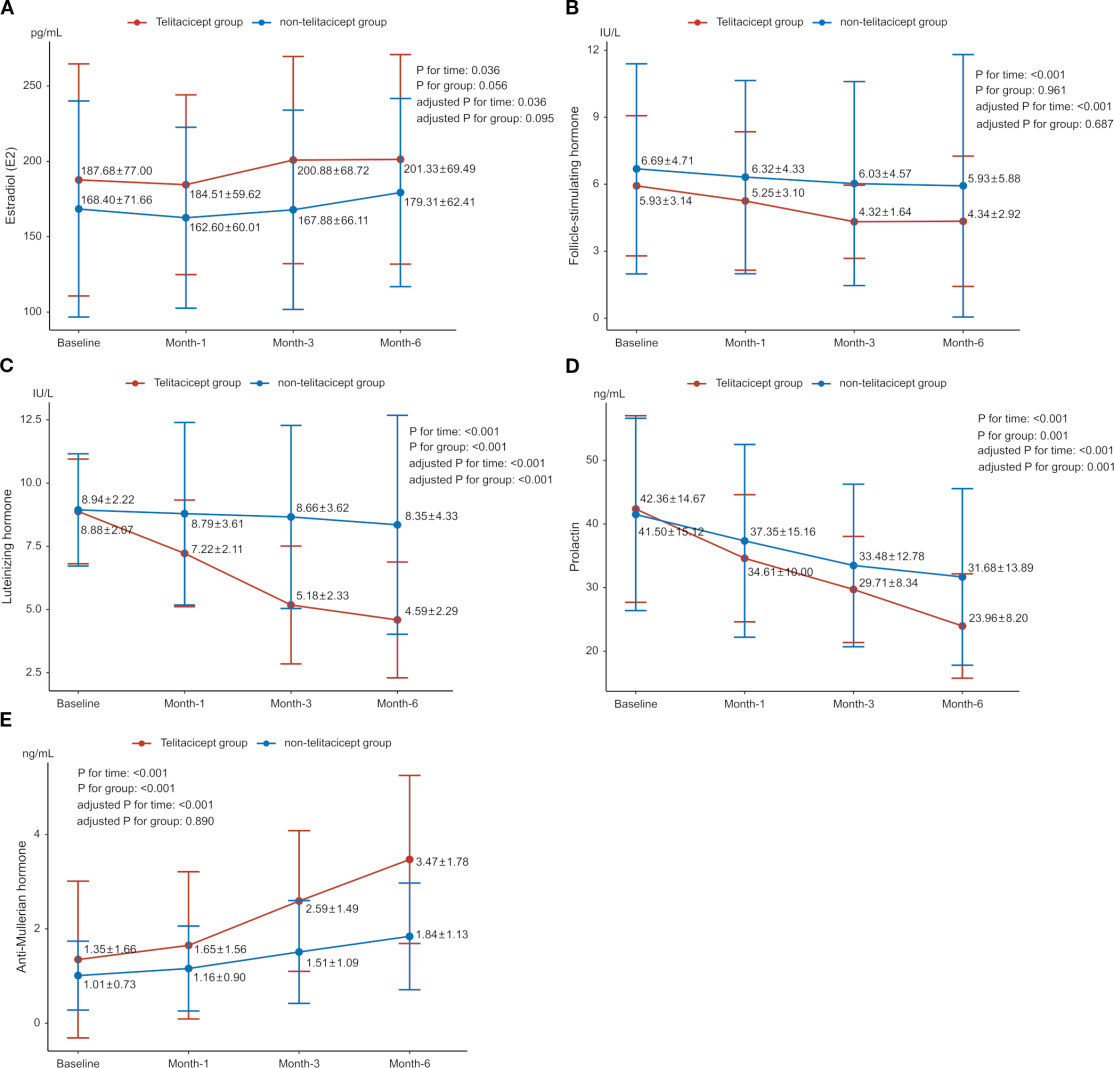
Changes in sex hormone levels between the telitacicept and non-telitacicept groups over time. **(A)** Estradiol (E2); **(B)** Follicle-stimulating hormone (FSH); **(C)** Luteinizing hormone (LH); **(D)** Prolactin (PRL); **(E)** Anti-Müllerian hormone (AMH).

Baseline E2 levels were 187.68 ± 77.00 pg/mL in the telitacicept group and 168.40 ± 71.66 pg/mL in the non-telitacicept group (P = 0.447). E2 levels increased significantly over time (P_time_=0.036, adjusted P_time_=0.036), primarily driven by a significant difference between 1 month and 6 months in the non-telitacicept group (162.60 ± 60.01 pg/mL *vs*. 179.31 ± 62.41 pg/mL, Bonferroni corrected P = 0.012). Despite these changes, there was no significant difference in E2 levels between the two groups over the entire study period in GEE analysis (P_group_=0.056, adjusted P_group_=0.095). Between-group comparisons at different time points indicated that at 1 month and 3 months post-treatment, E2 levels in the telitacicept group were significantly higher than in the non-telitacicept group (month 1: 184.51 ± 59.62 pg/mL *vs*. 162.60 ± 60.01 pg/mL, P = 0.016; month 3: 200.88 ± 68.72 pg/mL *vs*. 167.88 ± 66.11 pg/mL, P = 0.007), with no significant difference was observed at month 6 post-treatment (P = 0.108) (**Figure 1A**).

The baseline FSH levels were 5.93 ± 3.14 IU/L and 6.69 ± 4.71 IU/L in the telitacicept and non-telitacicept group (P = 0.285), respectively. FSH levels decreased significantly over time in the GEE analysis (P_time_<0.001, adjusted P_time_<0.001). In the telitacicept group, FSH levels significantly decreased from baseline at month 1, 3 and 6 (Bonferroni corrected P = 0.002, 0.001, and <0.001, respectively). However, there was no significant difference between the two groups over time in GEE analysis (P_group_=0.961, adjusted P_group_=0.687). Between-group comparisons at different time points revealed that at 3 months and 6 months post-treatment, FSH levels in the telitacicept group were significantly lower than in the non-telitacicept group (month 3: 4.32 ± 1.64 IU/L *vs*. 6.03 ± 4.57 IU/L, P = 0.006; month 6: 4.34 ± 2.92 IU/L *vs*. 5.93 ± 5.88 IU/L, P = 0.006) (**Figure 1B**).

For LH, baseline levels were 8.88 ± 2.07 IU/L in the telitacicept group and 8.94 ± 2.22 IU/L in the non-telitacicept group (P = 0.899). LH levels decreased significantly over time (P_time_<0.001, adjusted P_time_<0.001). This significant change was primarily due to the substantial decrease in LH levels in the telitacicept group from baseline at month 1, 3 and 6 (all Bonferroni corrected P<0.001). Overall, LH levels in the telitacicept group were significantly lower than those in the non-telitacicept group over the study period (P_group_<0.001, adjusted P_group_<0.001). Between-group comparisons at different time points revealed that LH levels in the telitacicept group were significantly lower than in the non-telitacicept group (month 1: 7.22 ± 2.11 IU/L *vs*. 8.79 ± 3.61 IU/L, P = 0.012; month 3: 5.18 ± 2.33 IU/L *vs*. 8.66 ± 3.62 IU/L, P<0.001; month 6: 4.59 ± 2.29 IU/L *vs*. 8.35 ± 4.33 IU/L, P<0.001) (**Figure 1C**).

The baseline PRL levels were 42.36 ± 14.67 ng/mL in the telitacicept group and 41.50 ± 15.12 ng/mL in the non-telitacicept group (P = 0.646). PRL levels decreased significantly over time in the two groups (P_time_<0.001, adjusted P_time_<0.001) with significant decreases in PRL levels from baseline at month 1, 3, and 6 post-treatment were observed (all Bonferroni corrected P<0.05). Overall, PRL levels in the telitacicept group were significantly lower than in the non-telitacicept group (P_group_=0.001, adjusted P_group_=0.001). Between-group comparisons at different time points revealed that at 6 months post-treatment, PRL levels in the telitacicept group were significantly lower than in the non-telitacicept group (23.96 ± 8.20 ng/mL *vs*. 31.68 ± 13.89 ng/mL, P = 0.004) (**Figure 1D**).

The baseline AMH levels were 1.35 ± 1.66 ng/mL in the telitacicept group and 1.01 ± 0.73 ng/mL in the non-telitacicept group (P = 0.542). AMH levels increased significantly over time in both groups (P_time_<0.001, adjusted P_time_<0.001). The telitacicept group showed a significant increase in AMH levels when comparing 1-, 3-, and 6-month post-treatment to baseline (all Bonferroni corrected P<0.05). Overall, AMH levels in the telitacicept group were higher than in the non-telitacicept group throughout the study period (P_group_<0.001), opposite to the results after adjusting confounders (adjusted P_group_=0.890). Between-group comparisons at different time points revealed that AMH levels in the telitacicept group were significantly higher than in the non-telitacicept group at 1 month, 3 months, and 6 months post-treatment (month 1: 1.65 ± 1.56 ng/mL *vs*. 1.16 ± 0.90 ng/mL, P = 0.038; month 3: 2.59 ± 1.49 ng/mL *vs*. 1.51 ± 1.09 ng/mL, P<0.001; month 6: 3.47 ± 1.78 ng/mL *vs*. 1.84 ± 1.13 ng/mL, P<0.001) (**Figure 1E**).

**Figure 2:**
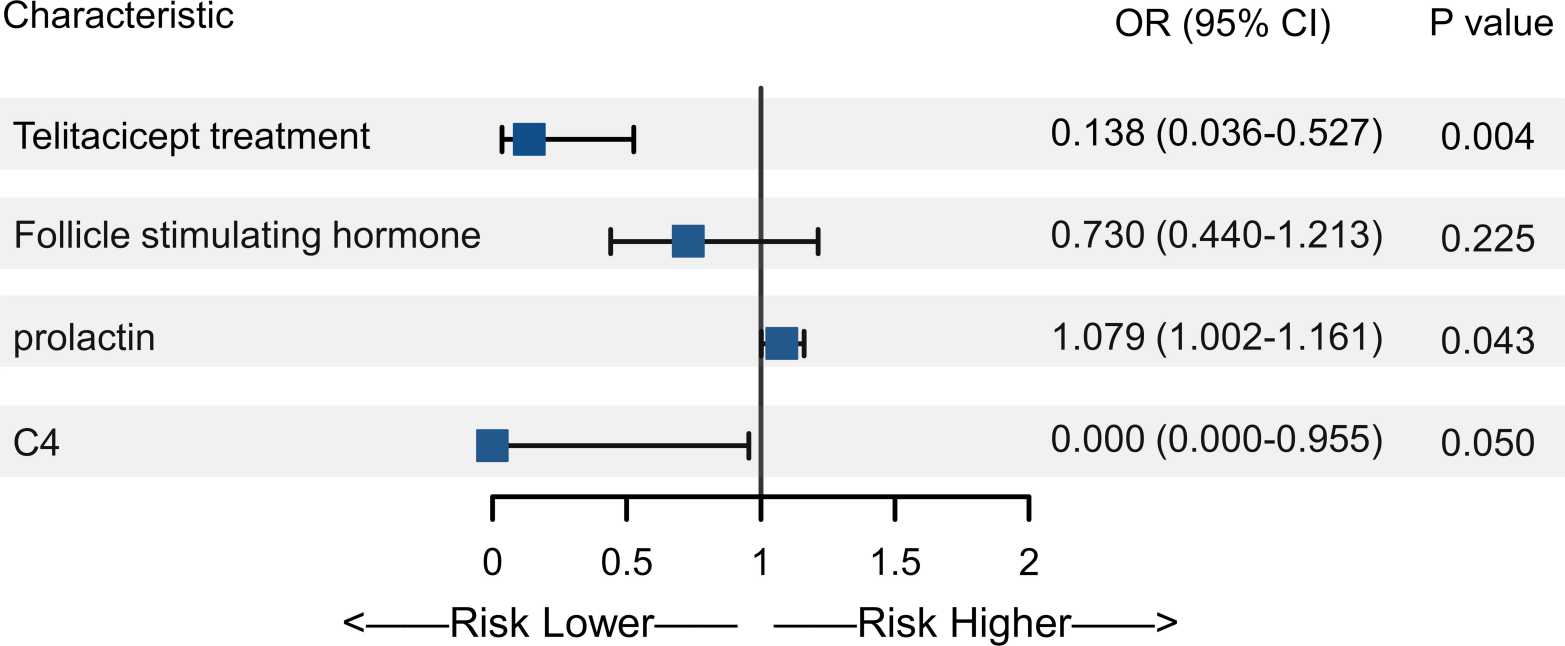
Forest plot of multivariate analysis of abnormal prolactin (PRL) levels. The forest plot displays the odds ratios (OR) and 95% confidence intervals (CI) for the variables included in the multivariate analysis.

Based on the normal value cutoffs, the numbers of patients with abnormal levels of E2, FSH, LH, PRL, and AMH after 6 months of treatment were 4 (5.1%), 12 (15.4%), 5 (6.4%), 45 (57.7%), and 12 (15.4%), respectively. With enough sample size for multivariate analysis, we only performed the analysis of associated with abnormal PRL level. In the univariate analysis, treatment with telitacicept, baseline E2, FSH, LH and PRL levels, baseline complement C3, C4 and IgG levels and glucocorticoid dose was associated with abnormal PRL level (all P<0.05) (**Table 2**). In the multivariate analysis, after adjusting the confounders (baseline FSH, PRL and C4 levels), telitacicept treatment was associated with a significantly lower incidence of abnormal PRL levels compared to the non-telitacicept treatment group (odds ratio [OR]=0.138, 95% confidence interval [CI]: 0.036-0.527, P = 0.004) (**Table 2**, **Figure 2**).

**Table 2:** Univariate and multivariate analysis of abnormal PRL.

Variables	Univariate analysis		Multivariate analysis	
	OR (95% CI)	*P*	OR (95% CI)	*P*
Telitacicept treatment	0.347 (0.137-0.880)	0.026	0.138 (0.036-0.527)	0.004
Age ≥30	1.371 (0.557-3.378)	0.492		
Disease course ≥175.5 days	1.371 (0.557-3.378)	0.492		
Menarche age	1.069 (0.747-1.528)	0.716		
Previous miscarriages	2.255 (0.806-6.313)	0.122		
Previous pregnancies	2.051 (0.801-5.255)	0.134		
Baseline E2	1.011 (1.004-1.019)	0.003		
Baseline FSH	0.663 (0.496-0.887)	0.006	0.730 (0.440-1.213)	0.225
Baseline LH	0.796 (0.632-1.004)	0.054		
Baseline PRL	1.113 (1.050-1.181)	<0.001	1.079 (1.002-1.161)	0.043
Baseline AMH	0.677 (0.408-1.124)	0.132		
Baseline SLE-DAI	1.262 (0.808-1.971)	0.306		
Baseline dsDNA antibody+	0.897 (0.232-3.470)	0.874		
Decreased platelet	0.966 (0.379-2.461)	0.941		
Decreased WBC	0.930 (0.378-2.285)	0.874		
C3	0.072 (0.010-0.508)	0.008		
C4	0.000 (0.000-0.001)	0.010	0.000 (0.000-0.955)	0.050
IgG	1.226 (1.067-1.408)	0.004		
24-hour urine protein	0.257 (0.035-1.886)	0.181		
Glucocorticoid dose	0.877 (0.780-0.986)	0.028		
MMF dose	0.111 (0.010-1.302)	0.080		
Hydroxychloroquine dose	0.002 (0.000-3782.151)	0.404		

OR, odds ratio; CI, confidence interval; E2, estradiol; FSH, follicle-stimulating hormone; LH, luteinizing hormone; PRL, prolactin; AMH, anti-Müllerian hormone; SLE-DAI, Systemic Lupus Erythematosus Disease Activity Index; dsDNA, double-stranded DNA; WBC, white blood cells; C3, complement C3; C4, complement C4; IgG, immunoglobulin G; MMF, mycophenolate mofetil.

Annotation: C3: 0.7-1.4g/L, C4: 0.1-0.4g/L

### 3.3 Disease control and laboratory parameters

**Figure 3:**
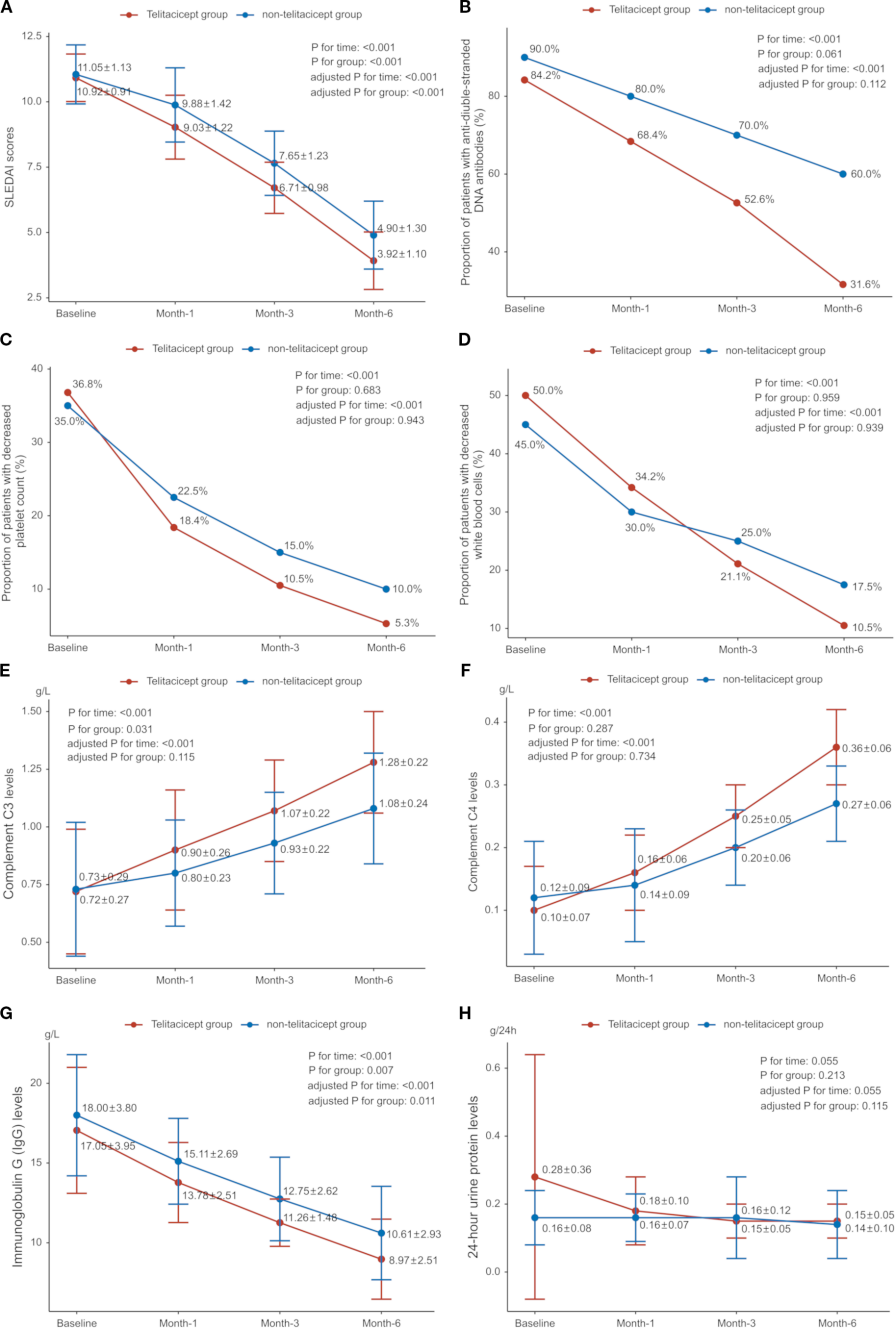
Disease control and change of laboratory parameters over time. **(A)** Systemic Lupus Erythematosus Disease Activity Index (SLEDAI); **(B)** Proportion of patients with anti-double-stranded DNA (anti-dsDNA) antibodies; **(C)** Proportion of patients with decreased platelet count; **(D)** Proportion of patients with decreased white blood cells (WBC); **(E)** Complement C3 levels; **(F)** Complement C4 levels; **(G)** Immunoglobulin G (IgG) levels; **(H)** 24-hour urine protein levels.

SLEDAI scores were 10.92 ± 0.91 in the telitacicept group and 11.05 ± 1.13 in the non-telitacicept group at baseline (P = 0.466). SLEDAI scores significantly decreased over time in both groups (P_time_<0.001, adjusted P_time_<0.001), with all Bonferroni corrected P<0.001. Notably, the telitacicept group had significantly lower SLEDAI scores throughout the study period compared to the non-telitacicept group (P_group_<0.001, adjusted P_group_<0.001). Between-group comparisons at different time points revealed that SLEDAI scores were significantly lower in the telitacicept group compared to the non-telitacicept group at month 1, 3, and 6 (month 1: 9.03 ± 1.22 *vs*. 9.88 ± 1.42, P = 0.002; month 3: 6.71 ± 0.98 *vs*. 7.65 ± 1.23, P<0.001; month 6: 3.92 ± 1.10 *vs*. 4.90 ± 1.30, P<0.001) (**Figure 3A**). After 6 months of treatment, a higher proportion of patients in the telitacicept group achieved SLEDAI scores ≤4 compared to the non-telitacicept group (73.7% *vs*. 40.0%, P = 0.003).

Complement C3 and C4 levels increased gradually in both groups, while immunoglobulin G (IgG) levels decreased (all P_time_<0.001, adjusted P_time_<0.001). IgG levels were significantly lower in the telitacicept group compared to the non-telitacicept group (P_group_=0.007, adjusted P_group_=0.011). There was a downward trend in 24-hour urinary protein in both groups (P_time_=0.055, adjusted P_time_=0.055). The proportion of patients with positive dsDNA antibodies decreased over time (P_time_<0.001, adjusted P_time_<0.001), but there was no significant difference between groups (P_group_=0.061, adjusted P_group_=0.112). Both groups showed significant declines in the proportion of patients with decreased platelet counts (both P_time_<0.001, adjusted P_time_<0.001), with no difference between groups (all P_group_>0.05). The between-group comparisons at different time point are presented in **Figures 3B–H**.

### 3.4 Concurrent treatment

The baseline doses of glucocorticoids were similar between the telitacicept and non-telitacicept groups (26.53 ± 4.58 mg/day *vs*. 25.48 ± 4.18 mg/day, P = 0.246). Both groups exhibited a significant reduction in glucocorticoid dosage at month 6 (P<0.001). Notably, the glucocorticoid dose at month 6 was significantly lower (6.80 ± 2.68 mg/day *vs*. 7.64 ± 1.97 mg/day, P = 0.003) and the proportion of patients received glucocorticoid doses ≤7.5 mg/day were higher (81.6% *vs*. 50.0%, P = 0.003) in the telitacicept group compared to the non-telitacicept group. At 6 months post-treatment, the MMF dose in the telitacicept group was significantly lower than in the non-telitacicept group (1.04 ± 0.27 g/day *vs*. 1.30 ± 0.25 g/day, P<0.001). However, no statistically significant difference was found in hydroxychloroquine doses between the two groups at 6 months post-treatment (0.35 ± 0.05 g/day *vs*. 0.36 ± 0.05 g/day, P = 0.381).

### 3.5 Safety

There was no significant difference in the occurrence of any AEs between the telitacicept group (17 patients, 44.7%) and the non-telitacicept group (18 patients, 45.0%) (P = 0.981). The most common AE was upper respiratory tract infections, with an incidence of 18.4% (n=7) in the telitacicept group compared to 15.0% (n=6) in the non-telitacicept group (P = 0.685). The incidences of gastrointestinal reactions, urinary tract infections, liver function abnormalities, herpes zoster and conjunctivitis were similar between the two groups (all P>0.05) (**Table 3**).

**Table 3:** Safety.

Events, n (%)	Telitacicept (n=38)	Non-telitacicept (n=40)	*P*
Any adverse event	17 (44.7)	18 (45.0)	0.981
Upper respiratory tract infections	7 (18.4)	6 (15.0)	0.685
Gastrointestinal reactions	4 (10.5)	4 (10.0)	0.939
Urinary tract infections	3 (7.9)	5 (12.5)	0.503
Liver function abnormalities	2 (5.3)	1 (2.5)	0.526
Herpes zoster	1 (2.6)	1 (2.5)	0.971
Conjunctivitis	1 (2.6)	2 (5.0)	0.587

## 4 Discussion

In the light of possible reproductive benefit from telitacicept and to fill the evidence gap, we investigated the effect of telitacicept on the gonadal function in female patients. In the present study, SLE treatment significantly improve the gonadal function in female patients with SLE, manifesting as increased levels of AMH and E2 and decreased levels of LH, FSH, and PRL. Notably, telitacicept treatment had a better positive effect on ovarian reserve and hormonal balance, especially with a greater reduction in LH and PRL levels, compared to those without telitacicept treatment. The multivariate analysis supported telitacicept treatment was associated with lower risk of abnormal PRL levels. Regarding disease control, telitacicept effectively controlled disease activity, resulting in significantly lower SLEDAI scores, and lower doses of glucocorticoids and MMF compared to those without telitacicept. No addition safety signal was identified. These findings suggest telitacicept as a promising therapeutic option for female SLE patients, offering benefits in disease control and reproductive health.

SLE is a complex autoimmune disease with diverse clinical manifestations and intricate autoantibody profiles. Genetic, epigenetic, and environmental factors contribute to ovarian dysfunction in female patients with SLE (18). Studies have shown that the risk of premature ovarian failure and diminished ovarian reserve is significantly higher in female SLE patients compared to healthy women of the same age (18). SLE-related ovarian dysfunction may result from a complex immune network involving B and T cell interactions, autoantibody production, immune complex formation, inflammatory reactions, and cytokine release (19), which can disrupt the hypothalamus-pituitary-ovary axis and impair follicular development (20). Furthermore, high-titer organ-specific antibodies targeting ovarian function, such as anti-ovary antibodies, can lead to autoimmune oophoritis and reduced ovarian reserve (21). Lupus flares are also associated with hyperprolactinemia, which can affect ovulation and the immune system (22). Studies have documented significant decreases in E2 and progesterone, increases in FSH, LH, and PRL, and decreases in AMH in SLE patients (23–25). Our study demonstrates that SLE treatment, whether with or without telitacicept, significantly improves gonadal function by elevating E2 and AMH levels while reducing FSH, LH, and PRL levels. These changes suggest that the SLE treatment might mitigate the impact of SLE on gonadal function, which emphasis strongly on the importance of timely treatment and disease control.

Traditional treatments like corticosteroids and cyclophosphamide have been associated with detrimental effects on sex hormone levels. Corticosteroids have been linked to increased LH levels and bioactive testosterone deficiency in male SLE patients (11), while cyclophosphamide has been shown to significantly lower AMH levels in female patients (10, 12, 26), contributing to ovarian dysfunction and might lead to amenorrhea (27). In a meta-analysis, the cumulative dose of cyclophosphamide was associated with premature ovarian failure, while mycophenolate, azathioprine, calcineurin inhibitors and steroids had a lower risk (9). In contrast, telitacicept’s ability to modulate immune responses and reduce systemic inflammation likely underlies its beneficial effects on gonadal function. In the present study, while both improves gonadal function, telitacicept showed a significantly greater reduction in LH and PRL levels compared to non-telitacicept treatments, with a marked reduction in the incidence of abnormal PRL levels. In preclinical studies, PRL increases the cytotoxic activity of T lymphocytes and the secretion of proinflammatory cytokines, which plays crucial role in SLE pathogenesis (28, 29). By significantly reducing PRL levels and improving overall hormonal balance, telitacicept not only offers effective disease control but also improve reproductive health. In the meantime, the level of AMH was significantly higher with telitacicept compared to non-telitacicept treatment in between-group comparison and GEE analysis, while no significantly difference was observed after adjusting confounders, which might suggest the confounding effect of baseline AMH levels. The levels of E2 at month 6 were similar between the two groups, mostly within the normal range, which might attribute to the relatively small sample size, and possible treatment at a relatively early disease stage. Though these results underscore the potential of telitacicept as a promising therapeutic option for preserving gonadal function in female SLE patients, the disparity of findings among different hormones might suggest the complex mechanisms of action regarding reproductive function, which warranted further investigations.

In terms of controlling SLE disease activity, a high proportion of patients achieved an SLE Responder Index 4 (SRI-4) response in the phase 2b trial (15). The phase 3 trial further confirmed the efficacy of telitacicept in reducing disease activity, with sustained improvements in SLEDAI scores, complement levels, and immunoglobulin levels, and a favorable safety profile (16). Overall, our findings align with and extend the evidence from previous studies, reinforcing the potential of telitacicept as a robust treatment option for SLE. In the present study, telitacicept has demonstrated superior real-world effectiveness compared to non-telitacicept treatments, with significantly reduced SLEDAI scores and requiring smaller doses of corticosteroids and MMF than non-telitacicept therapies. In addition, the level of 24-hour urinary protein in the telitacicept group showed a downward trend to the threshold of negative results (below 0.16g/24h). Due to the numerically lower level of 24-hour urinary protein at baseline in the non-telitacicept group, the between-group difference was not observed. It offers enhanced disease control, improved hormonal balance, and reduced medication burden, making it a valuable addition to the treatment for SLE patients.

An assessment of the safety profile of telitacicept reveals no additional safety signals compared to non-telitacicept treatments. The incidence of AEs was similar between the telitacicept and non-telitacicept groups, with no statistically significant differences observed in the occurrence of upper respiratory tract infections, gastrointestinal reactions, urinary tract infections, liver function abnormalities, herpes zoster, or conjunctivitis. These findings are consistent with previous phase II and III studies, which also reported comparable safety profiles between telitacicept and placebo groups (15, 16). Thus, telitacicept presents a safety profile similar to standard treatments, reinforcing its viability as a therapeutic option for SLE without additional safety concerns.

Our study has several limitations that warrant consideration. Firstly, the small sample size may limit the generalizability of our findings. With a larger cohort, we could achieve more robust statistical power and a better understanding of the variability in response to telitacicept treatment among diverse patient populations. Secondly, the limited follow-up duration restricts our ability to assess the long-term effect on gonadal function, as well as the long-term effectiveness and safety of telitacicept. In addition, the analysis of clinical outcomes concerning gonadal function (e.g. abnormal hormone levels) were limited due to small sample size or short follow-up. Given that our study population included patients with active disease and potential gonadal impairment, fertility-related assessments (e.g., ovulation monitoring) were not performed as pregnancy would not be recommended under these clinical circumstances. Larger-scale studies with longer-term follow-up are ongoing to specifically evaluate the menstrual cyclicity, ovulation or reproductive outcomes in SLE patients receiving telitacicept, especially in those with well-controlled disease. Lastly, though potential confounding was adjusted using GEE or multivariable regression analyses, the possible disparity of baseline characteristics between groups emphasizes the need for cautious interpretation of findings and future validation in larger cohorts.

In conclusion, telitacicept significantly improves gonadal function in female patients with SLE, with increased E2 and AMH levels and reduced FSH, LH, and PRL levels. Greater reduction in LH and PRL levels was observed with telitacicept compared to those without telitacicept treatment, suggesting a better enhancement of ovarian reserve and hormonal balance. Telitacicept effectively controlled SLE disease activity, with favorable safety profile. These results support telitacicept as a promising treatment for SLE, offering benefits in both disease control and reproductive health.

## Data Availability

The original contributions presented in the study are included in the article/supplementary material. Further inquiries can be directed to the corresponding author.
